# Using the Internet to Cope With Emotional Problems: The Role of Preference of Online Communication in Adolescents Across 18 Countries

**DOI:** 10.1111/sjop.70103

**Published:** 2026-05-12

**Authors:** Anke Görzig, Marie Jaron Bedrosova, Raffaella M. Milani

**Affiliations:** ^1^ School of Human Sciences and Institute for Lifecourse Development University of Greenwich, Old Royal Naval College London UK; ^2^ Interdisciplinary Research Team on Internet and Society, Faculty of Social Studies Masaryk University Brno The Czech Republic; ^3^ Department of Psychology and Human Development, School of Childhood and Social Care University of East London London UK

**Keywords:** coping strategies, emotional problems, excessive internet use, preference for online communication

## Abstract

For adolescents with emotional problems, internet use can provide coping strategies. These can be maladaptive (e.g., excessive use) or adaptive (e.g., engaging in social online activities). However, little is known about the mechanisms underlying both positive and negative outcomes. This study investigates whether a preference of online communication mediates associations between emotional problems and online coping strategies. Survey data from 14,550 adolescents (ages 11–16, 51% female) across 18 European countries were analyzed using mediation models. Emotional problems were associated with both maladaptive and adaptive strategies, and these effects were partially mediated by preference of online communication. The findings provide further empirical support for the model of compensatory internet use among vulnerable populations and demonstrate that similar mechanisms may underlie seemingly divergent outcomes. Implications include digital mental health interventions tailored to vulnerable youth and guidance for schools, families, and online platform providers to foster adaptive internet use whilst mitigating the risks of excessive or maladaptive use.

## Summary

- Adolescents with emotional problems use the internet as a coping strategy in both adaptive and maladaptive ways.
- Excessive internet use is a maladaptive online coping strategy.
- Engaging with social online activities is an adaptive online coping strategy.
- Preference for online communication partially facilitates both types of online coping strategies among those with emotional problems.

## 1 Introduction

Internet use for social activities is highly prevalent among young people in Europe. Since 2010, the time that children and adolescents spend online daily has doubled in most European countries (Smahel et al. 2020). This often means that they have ‘anywhere, anytime’ connectivity to online social activities, with the majority reporting they use their smartphones ‘daily’ or ‘all the time’ for online communication (Smahel et al. 2020). These online social activities, such as social networking sites (SNS), video, and gaming sites, play a crucial role in the lives of many adolescents, the nature and frequency of which shapes their consequences, with both positive and negative outcomes reported (Baker and Algorta 2016; Boniel‐Nissim et al. 2024; Cerniglia et al. 2017; Fassi et al. 2025; Pagliaccio et al. 2024; Zhou and Cheng 2022).

Emotional problems, such as depression, low self‐esteem, loneliness, shyness, and anxiety, are increasingly common among young people and adolescents (Ma et al. 2021; Sacco et al. 2024). Internet use appears to be specifically high among young people experiencing these issues. It has been suggested that for adolescents with emotional problems, internet use serves as a coping strategy that can be maladaptive as well as adaptive (Fassi et al. 2025; Folkman and Moskowitz 2004; Kardefelt‐Winther 2014, 2016, 2017; Zhou and Cheng 2022). Lazarus and Folkman's (1984) theory of stress and coping put forward that coping involves (1) a stressor, (2) cognitive appraisal, and (3) coping strategies. Coping strategies are employed to reduce or manage stress and can be adaptive (e.g., positive reframing, seeking social support, use of emotional and instrumental support) or maladaptive (e.g., denial, substance use, disengagement; Folkman and Moskowitz 2004; Meyer 2001).

Moreover, those with emotional problems show a preference for online social interaction as this mode of communication provides greater control of their self‐presentation (Caplan 2010) and regulates psychological needs (Carpenter 2012; Vorderer et al. 2016). Although prior studies have examined either excessive use or social engagement online in relation to mental health (e.g., Boer et al. 2020; Lyyra et al. 2022; Soriano‐Molina et al. 2025; Zhou and Cheng 2022), few have explored both within an integrated framework—nor have they identified a shared psychological mechanism (e.g., preference of online communication) that may drive both outcomes. Addressing this gap, the present study investigates whether preference of online communication mediates the relationship between emotional problems and two distinct coping outcomes: excessive internet use (maladaptive) and social online activity (adaptive). Recognizing that adolescents often engage in both coping styles concurrently (e.g., Al‐Bahrani et al. 2013; Pagliaccio et al. 2024; Thompson et al. 2010; Whitman and Gottdiener 2015), we examine these pathways in parallel.

Therefore, the current research contributes to digital coping theory by advancing the compensatory internet use model (Kardefelt‐Winther 2014, 2016), showing that the same internal vulnerabilities (e.g., emotional problems and communication preferences) can give rise to both beneficial and harmful digital outcomes. Practically, findings have implications for mental health professionals, educators, and digital literacy programs aiming to support young people's online behavior in a rapidly evolving media landscape (e.g., Fassi et al. 2025; Pagliaccio et al. 2024; Radovic et al. 2017; Wiederhold 2020).

### 1.1 Excessive Internet Use as a Maladaptive Coping Strategy

Maladaptive coping behaviors involve temporarily relieving symptoms, without addressing the root cause of the problem. These coping strategies include avoidance behaviors like emotional numbing, disengagement, and self‐distraction (Compas et al. 2017; Genest et al. 1990; Meyer 2001). It has been put forward that excessive internet use is motivated by an attempt to alleviate or compensate for real life problems by those with emotional problems (Kardefelt‐Winther 2014, 2016). As such, internet usage can become a form of maladaptive coping strategy for those with emotional problems. Indeed, a recent meta‐analytic review shows a significant association between mental health problems, namely depression, anxiety, and suicidal behavior, and excessive SNS use of adolescents (Soriano‐Molina et al. 2025). Excessive or problematic internet use is present when a young person shows a cognitive preoccupation with the internet, an inability to control its use, going online to relieve emotional distress, and continued use despite negative consequences (Caplan 2010; Griffiths 2000). Examples of maladaptive internet use include behaviors like spending excessive hours online to escape problems, neglecting schoolwork or sleep, or feeling unable to control internet use despite its negative consequences (e.g., skipping meals or family conflicts due to internet overuse; Kardefelt‐Winther 2016). It is especially the latter, negative consequences, which make this behavior maladaptive.

The model of compensatory internet use suggests that those who use the internet excessively are motivated to do so in order to cope or compensate for emotional problems (Kardefelt‐Winther 2014, 2016). The model posits that excessive internet use stems from the need to escape from real life issues or to alleviate dysphoric moods. This can result in negative outcomes such as a loss of control over internet use to the point where the individual experiences problematic consequences which negatively affect their life (Young and de Abreu 2011). A qualitative investigation of European adolescents' internet use showed that increased internet use can become maladaptive, especially when it is connected to compensatory or escape‐driven motives, substituting offline sociability and causing alienation from close people and offline ties (Tzavela et al. 2015). Empirical research further bolsters the notion that excessive internet use can be a maladaptive coping mechanism for those with emotional problems (Fassi et al. 2025; Kardefelt‐Winther 2016; Kumar et al. 2025; Tsigilis 2019). Indeed, problematic or addictive patterns of online use—including pathological involvement with social networking sites—have been associated with emotional problems (Ceyhan and Ceyhan 2008; Fassi et al. 2025; Giota and Kleftaras 2013). In addition, young internet users high on traits such as social anxiety and loneliness have been found to be at an increased risk of neglecting schoolwork and having conflicts with their parents due to their excessive engagement with the internet (Fassi et al. 2025; Kardefelt‐Winther 2014; Tsigilis 2019).

### 1.2 Social Online Activities as an Adaptive Coping Strategy

The model of compensatory internet usage postulates that internet usage for those with emotional problems may also be an adaptive, in addition to a maladaptive, coping mechanism (Kardefelt‐Winther 2014, 2016, 2017). Adaptive coping strategies include positive reframing, seeking social support, and use of emotional and instrumental support (Folkman and Moskowitz 2004; Meyer 2001).

The compensatory paradigm therefore suggests that for those with emotional problems, internet usage may provide a positive outlet in the form of providing online social interaction and support that the individual may lack in their offline life (Kardefelt‐Winther 2016, 2017; Keles et al. 2020; Tzavela et al. 2015), for example, by easing communication or overcoming shyness. Adaptive internet use involves positive engagement, such as adolescents using social media to maintain friendships, join interest groups, or seek emotional support during stressful times (e.g., chatting with friends about personal issues or participating in online hobby groups; Kumar et al. 2025; Zhou and Cheng 2022). Further, adaptive internet use can also happen when online interactions bridge or continue offline ties, rather than replace them (Hamilton et al. 2023; Tzavela et al. 2015).

There are several positive outcomes associated with internet use for social activities (Cerniglia et al. 2017; Vossen and Valkenburg 2016). For instance, young people can stay connected to each other, enabling them to find support online that may be lacking in traditional relationships (Akram and Kumar 2017; Weinstein 2018), as well as accessing advice and information (Siddiqui and Singh 2016) that may help them address psychological problems such as social anxiety and depression (Keles et al. 2020). For example, it has been posited that online social activities played a role in alleviating mental health problems such as anxiety and depression through promoting connectivity during the COVID‐19 pandemic (Ellis et al. 2020; Gao et al. 2020; Hamilton et al. 2023; Wiederhold 2020). Hence, those whose offline lives are characterized by a lack of social interaction might be motivated to go online to socialize, and this increased socialization may have positive effects on self‐esteem and psychological well‐being (Caplan et al. 2009; Kardefelt‐Winther 2017; Zhou and Cheng 2022). Thus, in these cases, social online activities can be seen as an adaptive coping mechanism (Kardefelt‐Winther 2016, 2017; Radovic et al. 2017; Weinstein 2018).

Indeed, empirical evidence confirms that internet use can be an adaptive coping mechanism for those with emotional problems. For instance, lower levels of depression have been shown to result from receiving social support and feeling socially connected via Facebook (Grieve et al. 2013; Wright et al. 2013) or from engaging in positive social comparison online (Lup et al. 2015). Socially rejected children engaged in social media as a positive coping mechanism by sharing their personal difficulties (Ophir 2017). Furthermore, online social networking has been shown to contribute as a mental health resource or intervention (Keles et al. 2020). Accordingly, it has been postulated that for depressed adolescents, social media may act as an adaptive coping mechanism (Masedu et al. 2014; Zhou and Cheng 2022) by enabling them to find connections and acceptance that they lack offline. In addition, Radovic et al. (2017) also demonstrated that adolescents suffering from depression were able to find social support through online communities by connecting with others experiencing similar issues.

Consolidating the notions that excessive internet use tends to be maladaptive, while social online engagement can be beneficial, recent large‐scale cross‐national studies across 29–42 countries have shown that adolescents exhibiting symptoms of social media addiction—akin to excessive use—reported the poorest mental and social well‐being (Boer et al. 2020; Boniel‐Nissim et al. 2022). In contrast, those who engaged intensively in social contact online experienced greater peer support (Boer et al. 2020) and the most positive mental and social well‐being outcomes (Boer et al. 2020; Boniel‐Nissim et al. 2022). However, it should be noted that some benefits of intense social use varied across countries (Boer et al. 2020).

### 1.3 Preference of Online Communication as an Underlying Mechanism

A preference of online communication has been defined as ‘whether children find online communication easier and tend to self‐disclose more online than offline’ (Smahel et al. 2020, 100). Caplan (2003) further states that ‘preference for online social interaction is a cognitive individual‐difference construct characterized by beliefs that one is safer, more efficacious, more confident, and more comfortable with online interpersonal interactions and relationships than with traditional [face‐to‐face] social activities’ (p. 629). Caplan's work (2003, 2010) suggests that online as opposed to face‐to‐face interaction is perceived as less threatening and can enhance social efficacy for those with higher emotional difficulties (e.g., social anxiety, loneliness, differences in social skills). According to this theory, individuals with emotional difficulties may prefer online communication to seek social support, comfort, and companionship for mood regulation. However, whilst such mood regulation may increase positive affect and other beneficial outcomes such as social competence and self‐esteem, it may also lead to negative consequences via compulsive use and cause detriments in other areas (e.g., neglect of schoolwork, family, friendships; Caplan 2010). A preference of online communication may therefore facilitate both maladaptive as well as adaptive coping strategies.

Preference of online communication may be positive if children use the internet for identity exploration or for communication about sensitive topics that they may not otherwise be able to discuss. In these cases, online communication serves as a safe platform for natural exploration that is part of the child's development (Caplan et al. 2009). For young people with emotional problems, online communication is often preferred, as the online environment allows for better self‐control and self‐presentation (Caplan 2003; Leung 2011; Ye and Lin 2015) and helps regulate psychological needs related to social control, social connectivity, and belonging (Carpenter 2012; Vorderer et al. 2016), which can adaptively foster and sustain relationships (Simoncic et al. 2014).

Although such a preference can be beneficial since it enriches social life, it may facilitate problematic use or maladaptive coping strategies if it becomes central for communication, diminishing offline communication and relationships (Caplan et al. 2009; Lyyra et al. 2022). Accordingly, in an updated cognitive‐behavioral model of problematic internet use by Caplan (2003, 2010, based on Davis 2001), a preference for online social interaction is proposed as a cognitive component of problematic internet use. Indeed, it is suggested that compulsive internet usage is facilitated by a preference for online social interaction, especially in children and adolescents with emotional problems (Caplan 2003; Fassi et al. 2025; Leung 2011; Mikuška et al. 2020; Ye and Lin 2015).

When online interaction substitutes offline relationships rather than complementing them, negative outcomes can arise. Yoo and Jeong (2017) observed “a vicious circle” in their longitudinal study of SNS use among people with low social capital and depression: increased SNS use worsened depression, which in turn led to even more SNS use. This maladaptive coping can then further deteriorate social capital and relationships (Caplan et al. 2009). Furthermore, people with low self‐esteem or internalizing problems may not benefit socially, as they tend to post more negative content and receive undesirable responses (Forest and Wood 2012) or engage in more social comparisons and feedback monitoring, potentially harming their well‐being (Fassi et al. 2025; Radovic et al. 2017).

In conclusion, a preference for online communication and disclosure can have both positive and negative effects for children and adolescents with emotional problems. In the positive form, the online environment provides the means to enrich and supplement offline communication. In the negative form, the online environment substitutes for offline communication. A preference for online communication may therefore facilitate both adaptive as well as maladaptive coping strategies for adolescents with emotional problems.

While a preference of online communication plays a role, we recognize that various other factors influence whether adolescents with emotional difficulties engage in adaptive or maladaptive coping online. Motivations may include stress relief, community building, and identity exploration which are especially salient for those from minority backgrounds or vulnerable youth (Craig et al. 2021; Pagliaccio et al. 2024; Zhou and Cheng 2022). However, examining other contributing factors is beyond the scope of this study. By focusing on the preference of online communication as the mediating mechanism, our study contributes to clarifying how this specific factor may channel emotional problems into different forms of online coping, offering insights that can guide targeted prevention and intervention efforts.

Using a representative cross‐national sample from the EU Kids Online (EUKO) IV survey (Smahel et al. 2020), the present study seeks to investigate the relationship between emotional problems and both maladaptive as well as adaptive coping strategies online. It is proposed that emotional difficulties are associated with a preference for online communication, which may simultaneously facilitate maladaptive coping strategies via excessive internet use and adaptive coping strategies via increased social interaction. It is hypothesized that preference for online communication mediates between emotional problems and maladaptive as well as adaptive coping strategies. Due to the potential overlap and joint variance between the two types of coping strategies, they will be investigated in separate models to determine their individual impacts without controlling for one another.

The hypotheses are as follows:

Model 1 *Maladaptive coping*.

*Emotional problems are associated with maladaptive coping strategies in the form of excessive internet use*.

*Emotional problems are associated with preference of online communication*.

*The relationship between emotional problems and maladaptive coping strategies is mediated by preference of online communication*.

Model 2 *Adaptive coping*.

*Emotional problems are associated with adaptive coping strategies in the form of social online activities*.

*Emotional problems are associated with preference of online communication*.

*The relationship between emotional problems and adaptive coping strategies is mediated by preference of online communication*.

## 2 Materials and Methods

### 2.1 Participants and Procedure

The research uses data from the EUKO IV international survey which focuses on online practices, risk, and opportunities of European children and adolescents (Smahel et al. 2020). A sample of 14,550 children and adolescents from 18 European countries aged 11–16 years resulted from using listwise missing data exclusion. For more detailed country, age, and gender distribution, see Table 1. The questionnaire was translated into all national languages using the TRAPD method (Walde and Völlm 2023) with two translators and an adjudicator, ensuring cross‐cultural and linguistic equivalence across all languages. The translation was coordinated by expert members of the EUKO network within each country. To further support the validity of the questionnaire, cognitive testing was conducted in five participating countries to assess the translation, clarity, and ensure comprehension and participant understanding (Zlamal et al. 2020, 7).

**TABLE 1:** Age and gender distribution of the sample.

Country	*N*	*M*_age_	SD	Gender (% girls)
Croatia	654	13.53	1.65	46.9
Czechia	1949	13.67	1.68	50.8
Estonia	554	13.38	1.66	50.4
Finland	599	14.09	1.59	54.8
France	632	13.49	1.68	44.9
Germany	657	13.67	1.68	49.3
Italy	554	13.62	1.69	47.8
Lithuania	611	13.54	1.74	48.1
Malta	726	13.22	1.46	62.5
Norway	572	13.79	1.52	47.9
Poland	597	13.11	1.55	55.6
Portugal	1012	13.91	1.28	51.0
Romania	446	13.57	1.72	51.8
Russia	1127	14.11	1.46	54.9
Serbia	715	13.76	1.74	55.7
Slovakia	536	13.70	1.72	53.7
Spain	2037	13.10	1.43	49.3
Switzerland	572	13.44	1.33	51.2
Total	14,550	13.58	1.60	51.4

*Note:* Listwise exclusion resulted in small but significant overrepresentations of older children (0.76 years, *t*(16882) = −21.02, *p* < 0.001) and girls (Phi = 0.038, *p* < 0.001).

Data collection took place from October 2017 to May 2018 using CASI/CAWI (computer‐assisted self‐interviewing and computer‐assisted web interviewing) method in nine countries, CAPI (computer‐assisted personal interviewing) method in five countries, and PAPI (pen‐and‐paper personal interviewing) method in four countries (for specific details, see the technical report; Zlamal et al. 2020). Sampling was carried out either through schools or in households, depending on the country. The general sampling criteria included the child's age, gender, region, and, where relevant, urban or rural residence, to approximate a representative sample in terms of the sociodemographic composition of the participating countries. Where recruitment took place via schools, schools or classes for severe learning or physical disabilities were not included (for details, see Zlamal et al. 2020). Informed consent of participants and legal guardians was obtained. The presented sample was used in all analyses exclusive of weighting. Descriptive statistics of the representative sample with population weights as well as details about the ethics, data collection, and sampling methodology can be found in the EUKO IV technical report (Zlamal et al. 2020).

Data collection has been performed in accordance with the principles stated in the Declaration of Helsinki. The project followed national ethical guidelines, and ethical approvals were obtained in countries where required by national ethical bodies. Details about each participating country and the relevant bodies issuing ethical approval can be found in the technical report (Zlamal et al. 2020).

### 2.2 Measures

#### 2.2.1 Independent Variable

##### 2.2.1.1 Emotional Problems

Emotional problems were measured with four items (*α* = 0.78) (*I worry a lot*; *I am often unhappy, sad or tearful; I am nervous in certain new situations; I easily lose confidence; I have many fears and I am easily scared*) adapted from the Strengths and Difficulties Questionnaire (SDQ; Goodman et al. 1998). Respondents answered on a 4‐point scale from 1 (*not true for me*) to 4 (*very true for me*). This measure is routinely used for screening purposes by health services using cut‐off points; however, it was used as a continuous measure in the current study.

#### 2.2.2 Dependent Variables

##### 2.2.2.1 Maladaptive Coping Strategies (Excessive Internet Use)

Maladaptive coping strategies were assessed in the form of excessive internet use via the Excessive Internet Use scale (EUKO network; Smahel et al. 2020) based on Griffiths' criteria of excessive internet use (2000), including salience, mood modification, tolerance, withdrawal symptoms, and relapse. The scale was validated for children and adolescents in 25 European countries (Škařupová et al. 2015). It consisted of five items (*α* = 0.77) (*I have gone without eating or sleeping because of the internet*; *I have tried unsuccessfully to spend less time on the internet*; *I have felt bothered when I cannot be on the internet; I have spent less time than I should with either family, friends*, *or doing schoolwork because of the time I spent on the internet; I have caught myself using the internet although I'm not really interested*) and asked about the frequency of these experiences during the past year with a response scale from 1 (*never*) to 5 (*daily or almost daily*). In Lithuania, one item (*I have caught myself using the internet although I'm not really interested*) was not asked of 11–13‐year‐olds, for whom the mean scale was computed with the exception of this item.

##### 2.2.2.2 Adaptive Coping Strategies (Social Online Activities)

Adaptive coping strategies were assessed in the form of interpersonal activities which were selected from 15 online activities for children and adolescents developed by the EUKO network (Zlamal et al. 2020) based on the work by Helsper et al. (2015). Five items were used to include both online interaction with close offline as well as online ties (*α* = 0.65) (*I used the internet to talk to people from other countries*; *I discussed political or social problems with other people online; I visited a social networking site*; *I communicated with family or friends*; *I participated in an online group where people share my interests or hobbies*) and asked about the frequency of these activities during the past month on a scale from 1 (*never*) to 6 (*almost all the time*). The internal consistency of the social online activities scale (*α* = 0.65) reflects its multidimensional nature and is adequate given the scale's design, which was intended to capture distinct but theoretically related forms of online interaction (e.g., contact with friends, family, or people from other countries). As a result, high inter‐item correlations were not expected, yet it remains theoretically meaningful to treat the items as a single scale, as they collectively reflect the breadth of adolescents' online social engagement and form a coherent representation of adaptive coping strategies. This approach is consistent with prior research on adolescents' online activities (e.g., Boer et al. 2020). As Taber (2018) notes, when items measure unique facets of a broader construct, low inter‐item correlations or modest alpha values do not necessarily indicate poor measurement quality. Additional analyses of inter‐item correlations and factor loadings supported the scale's unidimensionality and adequate coherence.

#### 2.2.3 Mediator

##### 2.2.3.1 Preference of Online Communication

Three items (*α* = 0.71) based on the work by Smahel and colleagues (Smahel et al. 2012; Smahel et al. 2020) were used (*I find it easier to be myself online than when I am with people face‐to‐face*; *I talk about different things online than I do when speaking to people face‐to‐face*; *I talk about personal things online which I do not talk about with people face‐to‐face)*. The frequency of these experiences on a scale from 1 (*never*) to 4 (*always*) was assessed. One item (*I talk about personal things online which I do not talk about with people face‐to‐face*) was not asked in Romania; the mean scale was computed from the remaining items.

#### 2.2.4 Control Variables

##### 2.2.4.1 Sociodemographic Characteristics

Respondents were asked about their age and gender, scored 0 (*boys*) and 1 (*girls*). Countries of data collection were coded as dummy variables, with Czechia serving as the reference category and included as controls.

### 2.3 Data Analyses

Descriptive statistics were performed for all variables to determine means by country. Multiple regression‐based mediation analyses were performed using the PROCESS macro version 3.4 for SPSS 25 (Hayes 2018) applying 5000 bias‐corrected bootstrap samples. Two mediation analyses were conducted in three steps for maladaptive and adaptive coping strategies separately. In step 1 emotional problems were tested as a predictor for coping behaviors (i.e., excessive internet use for maladaptive coping strategies, social online activities for adaptive coping strategies). Step 2 ascertained whether the predictor (emotional problems) would be significantly associated with the mediator (preference of online communication). Finally, in step 3 the strength of the predictor (emotional problems) for coping behaviors was tested whilst controlling for the mediator (preference of online communication). It was further tested whether the association of emotional problems with coping behaviors decreased significantly between steps 1 and 3 (Hayes 2018). Participants' age, gender, and dummy coded country variables served as control variables. Complete case analyses without the use of weights were performed for all statistics. Given the cross‐sectional design of the EU Kids Online data, mediation analyses in this study do not imply causal relationships.

## 3 Results

### 3.1 Descriptive Statistics

All measures had total mean ratings below their midpoints and in none of the countries did the mean rating cross the midpoint (see Table 2). For descriptive statistics of the representative sample with population weights, the interested reader is referred to the technical report (Zlamal et al. 2020). Correlations between the main variables were moderate (see Table 3).

**TABLE 2:** Descriptive statistics for emotional problems, preference of online communication, social online activities and excessive internet use by country.

Country	Emotional problems^a^		Preference of online communication^a^		Excessive internet use^b^		Social online activities^c^	
	*M*	SD	*M*	SD	*M*	SD	*M*	SD
Croatia	1.64	0.66	1.88	0.85	1.88	0.93	2.60	0.83
Czechia	1.98	0.71	1.73	0.61	1.76	0.73	3.15	0.94
Estonia	1.78	0.65	1.83	0.69	1.75	0.70	2.86	0.82
Finland	1.83	0.83	1.96	0.73	1.70	0.68	2.37	0.76
France	1.76	0.72	1.94	0.78	1.79	0.78	2.48	1.05
Germany	1.59	0.55	1.82	0.73	1.62	0.67	2.13	0.79
Italy	1.70	0.64	1.62	0.69	1.47	0.59	2.59	0.81
Lithuania	1.81	0.72	2.01	0.79	1.53	0.62	3.00	0.89
Malta	2.18	0.84	1.92	0.74	1.92	0.81	2.88	0.96
Norway	1.69	0.70	1.83	0.64	1.74	0.70	2.86	0.77
Poland	1.87	0.78	1.71	0.68	1.80	0.79	2.65	0.88
Portugal	1.93	0.75	1.80	0.69	1.76	0.75	3.14	0.88
Romania	2.01	0.83	1.96	0.79	1.95	0.80	3.25	1.08
Russia	1.83	0.74	1.95	0.84	1.60	0.61	3.32	0.91
Serbia	1.80	0.74	1.61	0.63	1.75	0.78	2.95	0.75
Slovakia	1.57	0.62	1.77	0.70	1.38	0.62	2.65	0.84
Spain	2.08	0.73	1.65	0.69	1.68	0.69	2.57	0.91
Switzerland	1.79	0.70	1.68	0.65	1.87	0.81	2.62	0.89
Total	1.87	0.74	1.79	0.72	1.72	0.74	2.82	0.94

^a^ Scales: 1 to 4.

^b^ Scale: 1 to 5.

^c^ Scale: 1 to 6.

**TABLE 3:** Bivariate correlations between emotional problems, preference of online communication, social online activities and excessive internet use.

Variable	1.	2.	3.	4.
1. Emotional problems	1	—	—	—
2. Preference of online communication	0.18**	1	—	—
3. Excessive internet use	0.37**	0.27**	1	—
4. Social online activities	0.18**	0.30**	0.30**	1

** *p* < 0.01.

### 3.2 Mediation Analyses

#### 3.2.1 Maladaptive Coping Strategies (Excessive Internet Use)

**FIGURE 1:**
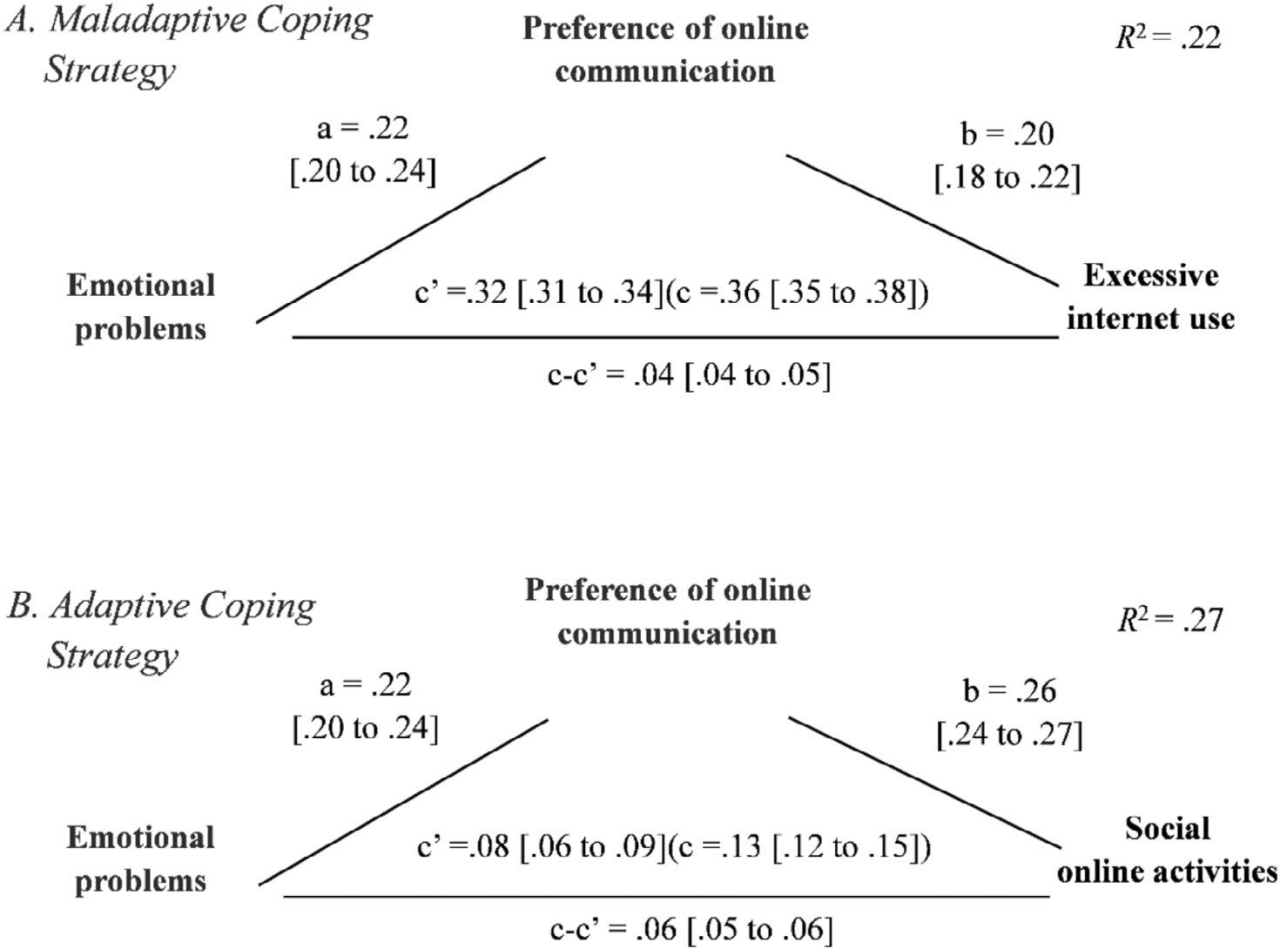
Standardized coefficients for mediational pathways of maladaptive (A) and adaptive (B) coping strategies. Numbers in squared brackets indicate 95% bootstrapped confidence intervals. *R* ^2^ indicates the explained variance for the regression model in step 3 (i.e., predictor, mediator and dependent variable). Control variables were: Participants' age, gender and dummy coded country variables.

Regression analyses confirmed that emotional problems significantly predicted excessive internet use (path c: *β* = 0.36, SE = 0.008) as well as preference of online communication (path a: *β* = 0.22, SE = 0.009). Emotional problems significantly predicted excessive internet use (path c': *β* = 0.32, SE = 0.008) whilst controlling for preference of online communication, which likewise significantly predicted excessive internet use (path b: *β* = 0.20, SE = 0.009). The indirect effect was significant albeit small (paths c–c': *β* = 0.04, SE = 0.003), indicating that a partial mediation occurs for the association between emotional problems and excessive internet use via preference of online communication (see Figure 1).

#### 3.2.2 Adaptive Coping Strategies (Social Online Activities)

Regression analyses confirmed that emotional problems significantly predicted social online activities (path c: *β* = 0.13, SE = 0.008) as well as preference of online communication (path a: *β* = 0.22, SE = 0.008). Emotional problems also significantly predicted social online activities (path c': *β* = 0.08, SE = 0.008) whilst controlling for preference of online communication, which likewise significantly predicted social online activities (path b: *β* = 0.26, SE = 0.008). The indirect effect was significant albeit small (paths c–c': *β* = 0.06, SE = 0.003), indicating that a partial mediation occurs for the association between emotional problems and social online activities via preference of online communication (see Figure 1).

## 4 Discussion

The present findings confirmed predictions for the association between emotional problems and adaptive coping strategies in the form of social online activities and for the association of emotional problems with maladaptive coping strategies in the form of excessive internet use. Findings further confirmed that both relationships were mediated by a preference of online communication; however, the mediational effects were relatively small.

Several studies have demonstrated a link between emotional problems or mental health difficulties and problematic internet use (Boer et al. 2020; Boniel‐Nissim et al. 2022; Ceyhan and Ceyhan 2008; Giota and Kleftaras 2013; Wegmann et al. 2015) to the detriment of offline activities and relationships (Kardefelt‐Winther 2014, 2016; Tsigilis 2019). The model of compensatory internet use proposes that excessive use is a result of a coping mechanism in the form of escapism or numbing for those with emotional problems; however, given the detrimental outcomes, it is considered maladaptive (Folkman and Moskowitz 2004; Kardefelt‐Winther 2016; Meyer 2001; Tsigilis 2019; Tzavela et al. 2015; Young and de Abreu 2011). The present findings confirm that engaging in excessive internet use increases with the level of emotional problems. Previous studies have also shown positive effects of internet or social networking use for individuals with mental health difficulties (Baker and Algorta 2016). Internet use may provide the social connectedness and support that those with emotional problems may need and may lack offline (Kardefelt‐Winther 2017; Keles et al. 2020). It has therefore been argued that online social networking can also provide a positive coping tool for those with mental health difficulties (Masedu et al. 2014; Zhou and Cheng 2022). For instance, marginalized children or those with emotional difficulties have benefited from creating social support and connections through social media (Grieve et al. 2013; Ophir 2017; Radovic et al. 2017; Wright et al. 2013). Social online activities, as measured in the current study (e.g., participating in a group with the same interests, communicating with family or friends), may promote social connectivity and support, thereby serving as an adaptive coping strategy for those with emotional problems (Folkman and Moskowitz 2004; Kardefelt‐Winther 2014, 2017; Meyer 2001). The present findings show that enhanced levels of emotional problems relate to an increased level of social online activities, suggesting that these may serve as an adaptive coping behavior.

Taken together, the present findings show that emotional problems are associated with both adaptive and maladaptive coping strategies, confirming the model of compensatory internet use which proposes that the internet can serve as a coping tool for mental health difficulties which can be maladaptive and detrimental to offline relationships and activities but at the same time adaptive in terms of providing social connectedness and support (Kardefelt‐Winther 2014, 2017; Pagliaccio et al. 2024; Tzavela et al. 2015; Zhou and Cheng 2022).

Emotional problems were further associated with a preference for online communication, which in turn was related to both maladaptive and adaptive coping mechanisms. The present findings therefore suggest that adolescents with emotional problems may have a higher preference for communicating and disclosing themselves online rather than offline due to the enhanced regulation of the social environment these platforms provide (Leung 2011; Simoncic et al. 2014; Ye and Lin 2015). Whilst online self‐disclosure and speaking to online friends can be adaptive due to associated increased levels of support and social activities online and offline (Abeele et al. 2017; Best et al. 2014; Simoncic et al. 2014; Xie et al. 2018; Zhou and Cheng 2022), it has also shown to be detrimental and maladaptive if offline activities and relationships are neglected as a result of excessive use (Caplan 2003; Caplan et al. 2009; Gong et al. 2024; Kardefelt‐Winther 2014, 2016; Prievara et al. 2019). The present findings suggest that both adaptive coping strategies (via social online activities) as well as maladaptive coping strategies (via excessive internet use) are associated with a higher preference for online communication or disclosure.

Finally, adaptive and maladaptive coping strategies correlated positively with one another, which suggests that it may be the same youth who engage in both behaviors and for similar reasons (e.g., preference of online communication). Indeed, previous research suggests that users of online platforms who used the internet excessively to the detriment of their offline lives, did so due to a motivation to connect socially with other individuals online (Tsigilis 2019). However, the mediation effect of preference of online communication was small for both pathways—from emotional problems to maladaptive as well as to adaptive coping—despite moderate associations between emotional problems, preference of online communication, and both coping strategies. This suggests that while preference of online communication may shape how emotional problems influence online coping, it explains only a small part of this relationship. In practical terms, a quarter to a third of differences in the outcome variables could be explained by our model, that is, 22% of the variation in social online activities and 27% of the variation in excessive use were explained. This indicates that preference for online communication functions as one of several contributing mechanisms rather than a dominant driver of coping behavior. Although the mediation effect is modest at the individual level, even small shifts in coping‐related online behaviors can have meaningful public health implications when they occur across large populations. This is particularly relevant given both the widespread use of the internet among adolescents and the high prevalence of emotional problems and mental disorders, such as anxiety and depression, among youth. A recent meta‐analysis indicates that up to 15% of young people in Europe are affected by a mental disorder (Sacco et al. 2024). However, other factors—such as the quality of peer and family relationships, perceived social support, emotional regulation, digital skills, dissociation, or mindfulness (Elmquist and McLaughlin 2018; Fassi et al. 2025; Gioia et al. 2021; Helsper and Smahel 2020; Verrastro et al. 2024)—are also likely to play a role in shaping how adolescents with emotional problems use the internet to cope. For example, family, school, and peer support can buffer distress and loneliness, mitigating the negative effects of overuse while enhancing the benefits of social connection (Hamilton et al. 2023; Gong et al. 2024; Prievara et al. 2019; Khasmohammadi et al. 2020; Mikuška et al. 2020). Similarly, although higher digital literacy can foster positive social engagement among non‐vulnerable youth, some competencies may amplify overuse and adverse outcomes in psychologically vulnerable adolescents (Helsper and Smahel 2020; Wu et al. 2023).

### 4.1 Limitations

This study has several limitations. Firstly, the proposed mediation effects were weak, suggesting that other factors than the preference of online communication might be more influential. However, given the widespread engagement of adolescents in online communication, even small effects can have practical significance at the population level. Addressing the preference of online communication can therefore serve as an entry point for promoting more adaptive digital coping strategies, by helping adolescents reflect on their online behaviors whilst remaining mindful of potential influences on coping via other factors.

In addition, despite the weak mediation effect, preference of online communication had a moderate association with emotional problems and with both maladaptive and adaptive coping strategies. This indicates that there might be additional mechanisms for these effects, and that preference of online communication may be an outcome of emotional problems as well as a predictor of maladaptive and adaptive coping strategies in its own right (cf. Caplan 2003, 2010). Further, whilst the present research has investigated social online activities as an adaptive coping strategy and excessive internet use as a maladaptive coping strategy, a generalizability to all maladaptive and adaptive coping strategies online must be considered with caution.

Further limitations are the assumptions of the direction of the effects or causality for the mediation effect within this cross‐sectional study. Whilst emotional difficulties are theoretically more likely to lead to a stronger engagement with social online activities (adaptive coping strategy) rather than the other way around, the same cannot be said for the association of emotional problems with excessive internet use (maladaptive coping strategy). In fact, excessive internet use may lead to or exacerbate emotional problems and vice versa (Ceyhan and Ceyhan 2008; Giota and Kleftaras 2013; Kardefelt‐Winther 2016; Wegmann et al. 2015); hence, the direction of the effect must be interpreted with caution.

In addition, the generalizability of our sample is limited; by using listwise data exclusion for our analysis, for some involved countries, our analytical sample over‐represented older children and girls over boys. However, the gender differences were very small. Whilst the age differences showed medium statistical effect size, the actual difference was less than a year (0.76 years). The age difference might have resulted from the fact that younger children took more time to complete the whole questionnaire and more often were not able to provide answers to all our questions. Whilst taking into account that gender and age were controlled for in the analyses, our findings may show a minimal bias towards girls and older children.

### 4.2 Future Directions

Whilst the current findings have confirmed that young people with emotional problems engage online in maladaptive as well as adaptive coping strategies in the form of excessive internet use and online social activities, further research may explore other types of maladaptive and adaptive coping strategies online. Given the weak mediational effects, additional research should also investigate other factors that motivate youth to engage in online coping strategies besides the preference of online communication. Future studies could explore how other factors, such as social support and digital skills (Helsper and Smahel 2020; Mikuška et al. 2020), may be used for interventions for mitigating the risks of excessive internet use and supporting healthy online social connections in youth. Moreover, it has been suggested that online self‐promoting behaviors might be driven by an attempt to regulate psychological needs deficits (Carpenter 2012; Trepte and Reinecke 2013; Vorderer et al. 2016). Types of self‐promoting behaviors, as well as psychological needs deficits (e.g., social connectivity, sense of belonging, or ostracism), may be assessed as additional motivational factors (Zhou and Cheng 2022). Adolescence is a critical developmental period for identity formation and peer validation, and some online behaviors may be shaped by social metrics such as likes and shares, especially among those with emotional problems (Fassi et al. 2025). Though not the primary focus of this study, we acknowledge that such peer‐based mechanisms may contribute to emotional responses online and are important to explore in future work. Further, it would also be important to explore whether increased online social activity can translate into improved offline social life (Davis 2012; Hamilton et al. 2023).

In addition, it is important to explore other moderating factors for the association between emotional problems and online coping behaviors. For example, which children under what circumstances are more likely to engage in maladaptive and/or adaptive coping strategies on the internet. A range of social and demographic factors, specifically those associated with social disadvantage or social capital, may be considered here (Ellison et al. 2007; Fujiwara and Kawachi 2008; van Deursen and Helsper 2015; Williams 2006). Such an approach would help to develop more targeted intervention and prevention strategies.

Future research should also adopt a developmental perspective. Age and gender have been shown to influence adolescents' online activities (Smahel et al. 2020), coping strategies (Vandoninck and d'Haenens 2015), and excessive internet use (Boniel‐Nissim et al. 2024). Younger adolescents tend to use the internet for entertainment and exploration, while older teens focus more on identity‐related and complex social interactions (Borca et al. 2015). Moreover, longitudinal findings point to developmental and gender‐specific windows of sensitivity to social media's impact on well‐being (Orben et al. 2022). These developmental differences could affect the balance between adaptive and maladaptive coping, suggesting that age‐specific or gender‐specific guidance might be necessary.

Our study focused on individual predictors and not on country contexts. However, differences for general coping styles have been found in Western and Eastern/Southern countries (Persike and Seiffge‐Krenke 2012; Seiffge‐Krenke et al. 2012). Although no cross‐national differences were hypothesized in our study, due to the relatively homogenous European cultural context compared to a global perspective, cultural context likely influences also how adolescents use the internet for coping. For instance, individuals in countries with stronger offline social support networks tend to use social media more for social connection, which can serve as an adaptive coping strategy that helps mitigate emotional problems. In contrast, in more individualistic societies, social media use often centers on entertainment or self‐presentation, which has shown weaker links to psychological benefits (Yin et al. 2019). Further, these benefits of intense social use varied across countries, such that intense social use was beneficial for well‐being when country‐level intense social use was high but less beneficial when this was low, suggesting that these associations may be influenced by national and cultural norms (Boer et al. 2020). Future research focused on wider cross‐country comparison beyond a European sample would shed light on the possible role of cultural context in adaptive and maladaptive coping strategies.

Furthermore, to confirm causality of the proposed effects—an area where current research remains limited (Fassi et al. 2025; Odgers and Jensen 2020)—longitudinal as well as experimental research needs to be considered in the future. Longitudinal studies that track adolescents over time would be particularly valuable in uncovering how emotional problems and online coping strategies influence one another across developmental stages. Such designs can help determine the directionality of effects and identify potential periods of increased vulnerability or resilience (Orben et al. 2022).

Lastly, other contextual variables at the family and country levels might be of importance. For example, the quality of the family environment and relationships could affect adolescents' internet use and coping strategies (e.g., Rega et al. 2023), while country‐level socioeconomic conditions, particularly national wealth and inequalities in wealth, may influence internet access and, in turn, adolescents' exposure to online risks and opportunities (cf., Görzig et al. 2021). Future research should incorporate more family‐ and contextual‐level variables to better disentangle their role and clarify factors affecting adolescents' coping strategies.

## 5 Conclusion

The present findings have shown that adolescents with emotional problems tend to use the internet to engage in coping strategies, which can be maladaptive or adaptive. Hence, assertions that are often presented and amplified by the mass media focusing on the detrimental outcomes of adolescents' internet use need expansion. A more nuanced approach should be considered that acknowledges that internet use can be associated with both positive and negative outcomes simultaneously (Baker and Algorta 2016; Cerniglia et al. 2017; Pagliaccio et al. 2024; Smith and Livingstone 2017; Valkenburg et al. 2022). It emerged that it is those who are struggling offline that are seeking to use the internet as a coping mechanism for their challenges. It was further revealed that this is at least partially driven by their desire to interact online as well as disclose themselves to others, which they may struggle with face‐to‐face (Akram and Kumar 2017; Weinstein 2018).

The present findings provide an indication that adolescents who face challenges offline, such as emotional difficulties, may benefit from guidance and platforms that support their use of the internet as a positive coping tool to promote their social interaction and connectedness. This may involve supporting the transition from online social activities to healthy offline interactions and communication, thereby ensuring a balanced online and offline social life and enhancing mental well‐being (Hamilton et al. 2023; Williams 2006). At the same time, such tools should include mechanisms to prevent excessive use which may otherwise exacerbate their mental health problems and create further disadvantages in face‐to‐face interactions and activities. The findings highlight the need to equip educators, parents, mental health professionals, and platform designers with strategies to recognize and support digitally mediated emotional coping in youth. Schools may implement curricula that help students distinguish adaptive from maladaptive online coping behaviors, while parents could benefit from digital literacy workshops addressing emotional disclosure, privacy, and peer dynamics (Pagliaccio et al. 2024). Mental health services might incorporate digital tools—such as text‐based check‐ins, guided journaling apps, and moderated peer forums—into treatment plans (Hollis et al. 2017; Pelletier‐Baldelli et al. 2015; Radovic et al. 2017), alongside interventions targeting online social comparison and the emotional impact of social media feedback (e.g., ‘likes’; Fassi et al. 2025). Additionally, platform designers could embed features such as time‐use nudges and distress detection tools for high‐risk youth (Fassi et al. 2025; Pagliaccio et al. 2024).

The present findings hold across age, gender, and country groups. Hence, findings are potentially relevant to a wide range of populations.

## Author Contributions

**Anke Görzig:** conceptualization, methodology, formal analysis, writing – original draft, writing – review and editing, visualization, project administration. **Marie Jaron Bedrosova:** methodology, formal analysis, writing – original draft, writing – review and editing, visualization; **Raffaella M. Milani:** conceptualization, formal analysis, writing – original draft, writing – review and editing. All authors approved the final version of the article.

## Funding

This work has been funded by a grant from the Programme Johannes Amos Comenius under the Ministry of Education, Youth and Sports of the Czech Republic from the project “Research of Excellence on Digital Technologies and Wellbeing CZ.02.01.01/00/22_008/0004583” which is co‐financed by the European Union.

## Ethics Statement

All data collection has been performed in accordance with the principles stated in the Declaration of Helsinki and participants provided informed consent prior to participation. Details of approvals can be found in the EUKO IV technical report (Zlamal et al. 2020).

## Conflicts of Interest

The authors declare no conflicts of interest.

## Data Availability

The data that support the findings of this study are available from the corresponding author upon reasonable request.
